# Relationships between body condition score and ultrasound skin-associated subcutaneous fat depth in equids

**DOI:** 10.1186/s13028-016-0243-2

**Published:** 2016-10-20

**Authors:** Severiano R. Silva, Rita Payan-Carreira, Miguel Quaresma, Cristina M. Guedes, Ana Sofia Santos

**Affiliations:** 1 CECAV-Centro de Ciência Animal e Veterinária, Universidade de Trás-os-Montes e Alto Douro, Quinta de Prados, 5000-801 Vila Real, Portugal; 2 EUVG-Escola Universitária Vasco da Gama, Campus Universitário, Bloco B, Lordemão, 3020-210 Coimbra, Portugal; 3 CITAB, Universidade de Trás-os-Montes e Alto Douro, Quinta de Prados, 5000-801 Vila Real, Portugal

**Keywords:** Adiposity, Subcutaneous fat, Ultrasound, Image analysis, Body condition, Obesity, Horses

## Abstract

**Background:**

In equids, health and welfare depend on body composition. A growing number of equids are now used as leisure and companion animals, and often found overfeed. The need for a close monitoring of body fatness led to the search for tools allowing a rapid and non-invasive estimation of fatness. This study intends to assess real-time ultrasonography (RTU) usefulness in establishing a relationship between ultrasound measures of subcutaneous fat–plus–skin thickness (SF-Skin) and body condition score (BCS) in horses and donkeys. Forty-three healthy animals (16 donkeys and 27 horses) were used in this study to generate 95 records (RTU and BCS pairs), in multiple RTU sessions for 2 years. Using visual appraisal and palpation, BCS was graded in a 1–9 points scale. Real-time ultrasonography images were taken using a 7.5 MHz linear transducer, placed perpendicular to the backbone, over the 3rd lumbar vertebra. ImageJ was used to measure the SF-Skin on RTU images. The relation between BCS and SF-Skin measurements was tested by linear and polynomial regression analysis.

**Results:**

The BCS values were similar in horses (5.50; from 3 to 8 points) and donkeys (5.14; from 3 to 7 points). The SF-Skin measures show a similar trend (a mean of 7.1 and 7.7 mm in horses and donkeys, respectively). A polynomial regression among BCS and SF-Skin explained 92 and 77 % of the variation in donkeys and horses respectively. The coefficient of determination was considerably higher for the regression developed for donkeys compared with that of horses (R2 = 0.92 vs. 0.77, respectively), which reduced the accuracy of the method in horses. Both the linear and polynomial models tested show a strong relationship among BCS and SF-Skin for donkeys (R2 > 0.91; P < 0.01) and horses (R2 > 0.74; P < 0.01), despite that the extremes for BCS did not existed in our sample.

**Conclusions:**

Our results showed the potential RTU usefulness to monitor body fat in equids. Using a high-frequency transducer and RTU together with image analysis allowed the identification of small SF-skin variations. This report will support further studies on the relationships between SF-Skin and BCS, particularly in extreme BCS scores.

## Findings

It is currently recognised that body fat is an important determinant of health status, productivity, reproductive efficiency and welfare in horses and donkeys [1, 2]. The body condition score (BCS) systems are currently used to assess body fat, though its ability to accurately reflect the adiposity in equine, has been challenged [3, 4]. Despite the simplicity in the use of body condition scoring, concerns were raised about the ability of untrained operators to accurately assess BCS [1, 5]. As a consequence, the search for tools allowing a rapid, non-invasive and inexpensive estimation of body composition is continuously pursued. Due to their body size, imaging technologies such as X-ray-computed tomography, dual-energy X-ray absorptiometry or magnetic resonance imaging, are of limited value for equids [3, 6]. Also the quantification of the equine body composition using gold standards—dissection and chemical analysis—is not practical as require intensive labour and the animals needs to be euthanized [3, 7]. Due to its operational characteristics, the real time ultrasonography (RTU), due to its operative characteristics, has been extensively used in farm animal management to assess body fat reserves [8–10] and was recently extended also to equids [2, 7]. RTU presents several advantages: offers a good spatial resolution, is reasonably priced, is well accepted and is easily performed on a standing animal under field conditions [2]. Data from multiple studies in different farm species [10–13] validated the precision of ultrasound by comparison to post-mortem subcutaneous fat measurements. However, RTU images require interpretation and sometimes the boundaries between tissues are not obvious [14, 15]. This is a problem that limits the accuracy of measurement, particularly in animals with thin subcutaneous fat deposits [16], which lead to the inclusion of the skin in the measures, reducing the error [12, 14]. Another important weakness is that the monitoring of subcutaneous fat is often limited to the evaluation of relatively superficial soft tissue structures with poor contrast; the use of a higher frequency transducer allows overcoming this issue [14, 17].

Nowadays, horses and donkeys are frequently kept for leisure and are not entirely dependent of the available forages in ranging systems, and tend to be overfed and often under-exercised. Thereby, an increased tendency for developing overweight or obesity has been reported, with adverse effects on animals health. As a consequence, it became of utmost importance to efficiently surveil the equids BCS. This work intends to establish a comprehensive relationship between ultrasound measures of subcutaneous fat–plus–skin thickness (SF-Skin) and body condition score (BCS) in horses and donkeys using RTU image analysis and a 7.5 MHz transducer.

This study enrolled 43 animals (16 Asinina de Miranda breed donkeys and 27 Garrano breed horses). Data was obtained in 10 different sessions (6 in donkeys and 4 in Garrano) during a 2-year period. A total of 95 records (53 representing donkeys and 42 horses) were obtained, comprising both the BCS evaluation and RTU image acquisition. No particular inclusion criteria were imposed, targeting the largest variation possible in animals BCS. All the animals were considered healthy after a physical examination, and pregnancy was discarded as the sole exclusion criteria of this study.

Donkeys belong to the local breed association (AEPGA–Associação para o Estudo e Protecção do Gado Asinino) while Garrano horses belonging to several owners and lived under traditional continuous grazing system in the region of Peneda-Gerês in the North of Portugal. Animals were kept under the natural photoperiod. Donkeys were maintained in a 2500 m^2^ paddock with an area of 50 m^2^ offering shelter from rain, sun and wind and were fed with 5–7 kg of hay and straw per jenny. During winter, 200–400 g of concentrate per jenny was also distributed twice daily. Clean fresh water was available ad libitum. Horses were kept on a traditional continuous grazing system based on natural pastures in free-ranging conditions. Concentrate supplement was offered to the animals when pasture availability was too low: either in summer and/or winter, depending on the climate conditions of the year.

The experiments were carried out in compliance with the Portuguese legal regulations for performing experiments on animals, with the owners’ informed consent, in accordance with the International Ethical standards.

Body condition was scored by two independent operators using the visual appraisal and hand-palpation in six areas of the body (the neck, the withers, the loin, the tail head, the ribs and the shoulders). A nine points scale system (1-very emaciated; 9-extremely fat) was used in horses and donkeys, as described by Henneke et al. [18], and Quaresma et al. [2], respectively. The final BCS value resulted from the average of the grades obtained in the six body parts.

RTU images were taken with an ultrasound scanner (Aloka SSD 500 V, Aloka Inc., Tokyo, Japan) using a linear 7.5 MHz transducer (UST-5512U-7.5, 38 mm, Tokyo, Japan). After a preliminary assay to select the most suitable spot to assess subcutaneous fat thickness, ultrasound scans were performed with the transducer placed at the animal´s back, over the 3rd lumbar vertebra, perpendicular to the backbone; all the images were collected on the left side of the animal.

The hair was trimmed at the measurement place and ultrasound gel (UltraPhonic, Codali, Newark, NJ, USA) was used as a coupling medium. The horses and donkeys were individually restrained during the ultrasound scanning to minimize movements and to ensure they were standing in a normal position.

RTU images were captured in video, for which the ultrasound scanner was connected to a video camera (DCR-HC96E, Sony, Tokyo, Japan). At the laboratory, videos were displayed and the most suitable images were saved in 720 × 576 TIFF image format and stored for posterior image analysis. To eliminate subjective operator-to-operator differences, image acquisition and measurements were done by only one experienced operator (SRS).

**Fig. 1:**
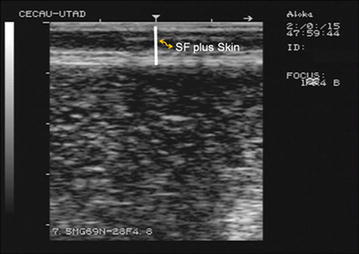
Example of a RTU image taken over the 3rd lumbar vertebra. A subcutaneous fat (SF) plus skin thickness is highlighted

Using ImageJ software (version 1.38×, National Institutes of Health, USA), RTU images were analysed to determine the SF-Skin measures (Fig. 1). Over each image, three SF-Skin measurements were obtained; the average of these three measures was used for data analysis.

All the statistical analyses were performed with the JMP software (version 7; SAS Institute, Cary, NC, USA). For the BCS and SF-Skin for both donkeys and horses, the basic descriptive statistics [mean, standard deviation, minimum and maximum values and coefficient of variation (CV)] were plotted. Regression analysis was performed to test the relationship between BCS and SF-Skin measurements, using linear and polynomial regression models. The coefficient of determination (R^2^) and the root mean square error (RMSE) were used to test the accuracy of the estimates [19].

Table 1 summarises the data on the BCS and SF-Skin of donkeys and horses. Body condition score and SF-Skin values were rather similar for both species. Body condition score in the horses enrolled in this study ranged from 3 to 8 points, with an average close to 5; their SF-skin values ranged from 3.33 to 12.65 mm, the average being set at 7.09 mm. Similarly, the average BCS in donkeys was 5.5, ranging from 3 to 7. The obtained SF-skin values ranged from 3.87 to 13.11 mm, with an average of 7.72 mm. In here, horses showed greater variation for BCS than donkeys (CV = 28.4 and 16.5 %, respectively), a trend also observed for SF-Skin measurements (CV = 25.0 and 30.9 %, for donkeys and horses respectively).

**Table 1:** Body condition score (BCS) and subcutaneous fat plus skin (SF-Skin) of donkeys (n = 53) and horses (n = 42)

BCS	Horses				Donkeys			
	N	Mean ± SD	Range	CV (%)	N	Mean ± SD	Range	CV (%)
3	3	3.71 ± 0.38	3.33–4.10	10.24	1	3.87	3.87	
4	9	5.49 ± 1.17	3.65–6.79	21.38	7	4.97 ± 0.66	4.25–5.67	13.29
5	6	6.39 ± 0.90	4.99–7.33	14.05	21	7.08 ± 0.74	5.78–8.33	10.45
6	10	6.76 ± 0.75	5.67–8.32	11.05	20	8.70 ± 0.68	7.69–9.98	7.81
7	7	8.23 ± 1.08	6.58–9.31	13.12	4	11.93 ± 1.33	10.31–13.11	11.18
8	7	10.48 ± 1.76	7.95–12.65	16.79				
SF-Skin^a^	42	7.09 ± 2.19	3.33–12.65	30.9	53	7.72 ± 1.93	3.87–13.11	25.0
BCS	42	5.50 ± 1.56	3.00–8.00	28.4	53	5.14 ± 0.85	3.00–7.00	16.5

Mean, standard deviation (±SD), range and coefficient of variation (CV)

^a^All SF-skin measures are in mm

**Fig. 2:**
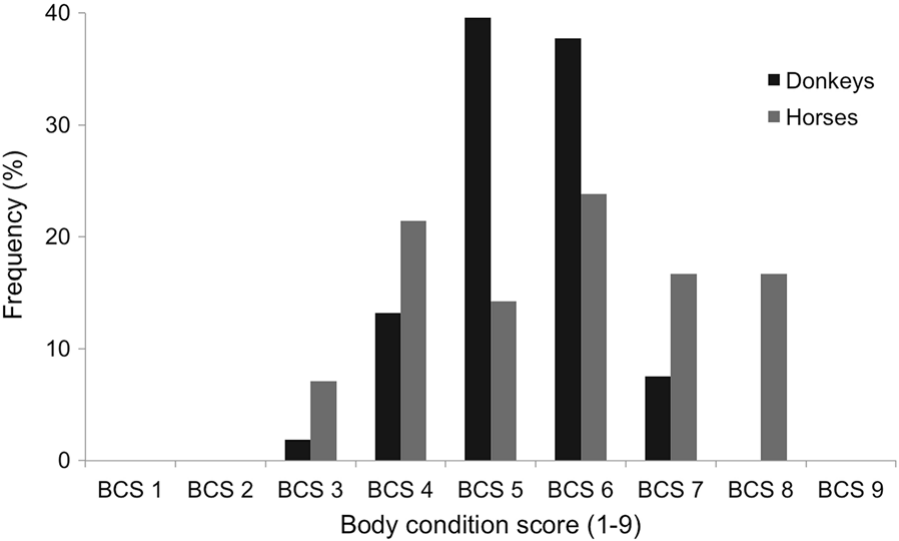
Histogram representing the frequency (percentage) body condition score class for donkeys and horses

Figure 2 shows a histogram representing the frequency (percentage) of the body condition score of donkeys and horses. For both species a reduced number of animals were observed in the BCS 3 class and the majority of animals (91 and 60 %, for donkeys and horses respectively) were found among BCS 4 and BCS 6 classes.

The evaluation of linear and polynomial relationship (R^2^ and RMSE) between BCS and SF-Skin measurements of donkeys and horses are presented in Table 2. The Linear and polynomial regressions are suited to explain (P < 0.001) the relationship between the BCS and SF-Skin by for both donkeys and horses. Both the models explained up to 70 and 90 % of the SF-Skin variation in horses and donkeys respectively. But the best fit was observed with the polynomial models that showed a RMSE of 0.648 and 1.063 mm for donkeys and horses respectively.

**Table 2:** Linear and polynomial relationship between body condition score (BCS) and subcutaneous fat and skin (SF-skin) measurements in donkeys (n = 53) and horses (n = 42)

Specie	Linear			Polynomial		
	R^2^	RMSE	P	R^2^	RMSE	P
Donkey	0.911	0.683	<0.001	0.921	0.648	<0.001
Horse	0.742	1.128	<0.001	0.772	1.063	<0.001

*R* ^*2*^ coefficient of determination; *RMSE* the root mean square error

**Fig. 3:**
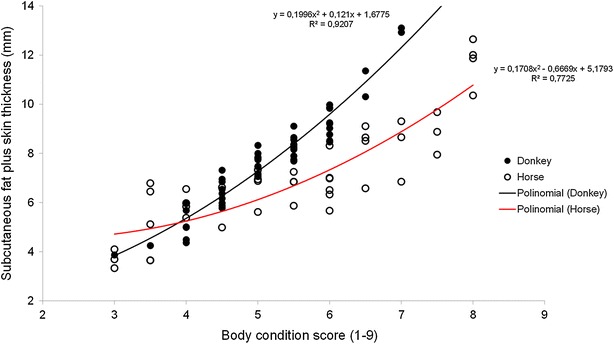
Polynomial regressions between subcutaneous fat plus skin thickness and body condition score. In donkeys (*black line* and *circles*) and in horses (*red line* and *open circles*)

The BCS and SF-Skin relationship were explained by a polynomial regression (Fig. 3). The coefficient of determination was considerably higher in the regression developed for donkeys than in the regression developed for horses (R^2^ = 0.92 vs. 0.77, respectively). These results correspond to an increased variation in SF-Skin within each score of body condition in horses compared to that of the donkeys.

For over 50 years, ultrasound techniques have been used in farm species to predict carcass composition; particularly, RTU has been confirmed in the last two decades as a valuable tool to predict and monitor body composition in living animals [10] with a few reports already existing in horses [7, 13, 20] and donkeys [2]. However, the use of RTU to assess fatness in equines is still limited, despite that the equine industry would greatly benefit from the development of imaging approaches capable of quantifying the body fat in living animals [3]. The present work could bring a significant contribution to better understand the relationship between BCS and the thickness of SF plus skin, in horses and donkeys. Data of BCS and SF plus skin using RTU were obtained over 2 years. It sought the widest possible range of BCS. Nevertheless, emaciated and obese animals were not easy to find, which limited the sample to animals with a BCS between 3 and 8. The average BCS was similar in horses and donkeys (5.50 and 5.14, respectively). It was akin to the BCS found in nonracing horses in Prince Edward Island [21], but lower than those reported by Dugdale et al. [22]. Moreover, in general, emaciated and extremely obese animals are cause for concern and corrective measures are introduced to mitigate the situation [23]. Although our sample don't include animals in the extremes of the body fat spectrum (BCS below 3 and above 8), the linear and polynomial models tested in the present study showed a strong relationship between BCS and SF-Skin for donkeys (R^2^ > 0.91; P < 0.01) and horses (R^2^ > 0.74; P < 0.01). This was supported by the results from other studies using ultrasound in horses, [13, 20, 24] which also report strong relationships between BCS and SF thickness (correlation coefficients (r) ranged from 0.64 to 0.92; P < 0.01) or donkeys [2] (r between 0.65 and 0.86, P < 0.01). For example, Gentry et al. [20] using ultrasonic fat measurements in mares at four different locations (tailhead, rump, 13th rib, and withers) found a stepwise regression analysis that explained 78 % of the BCS variation. Our results agree with those of Gentry et al. [20] and confirm the potential utility of the RTU to monitor fatness in horses.

Still, to monitor body fat over time by RTU implies the ability to identify small SF variations, for which the image analysis and a high frequency transducer may be helpful and very convenient. It has been suggested that the RTU accuracy for measure SF thickness (skin excluded) would be higher in fatter meat animals [16, 25], because of the error reduction associated with the ultrasound measurement determination. Similarly, in horses Kane et al. [26] refer that the estimation of fat thickness by ultrasonography is poorly correlated with the empty body fat when sites of lower fat thickness are used, due difficulties in their measurement. The increased difficulty to determine the smaller subcutaneous fat thickness and to clear identify the interface between the skin and subcutaneous fat led to the proposition to include the skin in SF measurement [27–29]. In the present study, the image analysis and the use of the 7.5 MHz transducer allowed obtaining SF measurements with a resolution of 0.2 mm. Usually, RTU equipment contain an internal measurement system which typically has a resolution of 1 mm [10] that, associated with the inclusion of skin on the RTU measurements, reduce errors in determining the SF in animals with reduced BCS or in longitudinal studies aiming to identify small variations in SF.

The nonlinear nature of the relationship between BCS and SF thickness showed that for the highest scores, one increment point on BCS translates into a more pronounced change in the SF-Skin measurement. This aspect was discussed by Argo et al. [3], based on the previous work of their team [4, 22], who defend that BCS may be a useful predictor of body fatness in animals in thin and moderate conditions but would not be a precise predictor of body fatness in overweight or obese individuals. This is also observed by Martin-Rosset et al. [30] who also found an exponential relationship between BCS and body fat content in 20 horses. Similar concerns have been raised in dairy cows and lambs [31, 32], which may limit the BCS usefulness as a toll to monitor is body fat variations in horses, particularly when fat content is too variable or in marks above 8 [22]. Dugdale et al. [22] stressed the need to identify more objective measurements of body fat in animals, especially for those in the moderate to the obese condition. Actually, adequate descriptors to distinguish the different levels of obesity of animals are lacking once skeletal landmarks become hidden by superficial adipose tissue [33]. However, that is not the case when using RTU. Furthermore, the differences in the accuracy given by the developed equations in the present study reinforce the need to establish models for each species separately.

This study proved the existence of a strong relationship between BCS and SF-Skin ultrasound measurement and showed that the RTU technique is able to measure variations of adiposity of equids. It also showed that species differences exist on the relationship between BCS and SF-Skin, as transposed to the linear and polynomial regressions generated in here. Future work enrolling a larger cohort of animals of different breeds and types, as well as including animals in the extreme BCS marks is recommend to sustain the results presented herein.

## Abbreviations

BCS: body condition score
CV: coefficient of variation
R^2^: coefficient of determination
RMSE: root mean square error
RTU: real-time ultrasonography
SF-Skin: subcutaneous fat–plus–skin thickness
